# Identification of EGFR expression status association with metastatic lymph node density (ND) by expression microarray analysis of advanced gastric cancer

**DOI:** 10.1002/cam4.311

**Published:** 2014-08-26

**Authors:** Akira Ema, Mina Waraya, Keishi Yamashita, Kenichi Kokubo, Hirosuke Kobayashi, Keika Hoshi, Yoshiko Shinkai, Hiroshi Kawamata, Kazunori Nakamura, Hiroshi Nishimiya, Natsuya Katada, Masahiko Watanabe

**Affiliations:** 1 Department of Surgery, Kitasato University School of MedicineKitasato 1-15-1, Minami-ku, Sagamihara, Kanagawa, 252-0374, Japan; 2 Department of Medical Engineering and Technology, Kitasato University School of Allied Health SciencesKitasato 1-15-1, Minami-ku, Sagamihara, Kanagawa, 252-0374, Japan; 3 Department of Preventive Medicine, Kitasato Clinical Research Center, Kitasato University School of MedicineKitasato 1-15-1, Minami-ku, Sagamihara, Kanagawa, 252-0374, Japan

**Keywords:** EGFR, gastric cancer, metastatic lymph node density, S-1 chemotherapy

## Abstract

Metastatic lymph node density (ND) has been reproducibly proven to be a prognostic factor in gastric cancer. The molecular mechanisms that underlie this aggressiveness are underexplored. Here, we aimed to identify molecules associated with this unique phenotype. Tumor specimens from patients with stage III gastric cancer with high or low ND (*n* = 4 for both) were compared at the mRNA level using Affymetrix microarray (harboring 54,675 genes). The expression data were prioritized, and genes that correlated with ND were selected. Ultimately, the *EGFR* was validated as such a candidate molecule in patients with primary advanced gastric cancer who underwent standard treatment (*n* = 167). Expression data of the microarray were prioritized based on gene expression ratio and frequency of gene expression. The first priority genes to be selected were genes that are known to be amplified in cancer, which included *NKX2.1, CHST9, CTNND2, SLC25A27, FGFR2, EGFR,* and *PTGER1*. Of these genes, the *EGFR* gene was of particular interest. EGFR expression in primary gastric cancer was examined using immunohistochemistry (IHC). The Student's *t*-test elucidated a significant difference in EGFR expression between IHC 2+/3+ and IHC 1+ according to ND (*P *=* *0.0035). The Chi-square test also indicated a significant difference between high and low levels of EGFR immunohistochemical staining (IHC2+/3+ and IHC1+, respectively) and ND status (*P *=* *0.0023). According to the least squares method, as ND increased, the risk that EGFR staining levels changed from IHC 1+ to IHC 2+ also increased. In this study, we determined that high EGFR expression may underlie the aggressive mechanism of advanced gastric cancer with high ND.

## Introduction

Gastric cancer is the second leading cause of cancer death worldwide 1. The prevalence of gastric cancer is very high in Japan, where approximately 110,000 people contract this disease each year, with 65,000 estimated deaths 2. The mainstay of treatment is curative surgery 3. However, many patients may have recurrence even after curative surgery. Various adjuvant chemotherapies have, therefore, been developed to prevent recurrence after surgery 4–6. S-1 is an oral therapeutic preparation that combines tegafur, gimeracil, and oteracil potassium. The prognostic benefit of S-1 was proven in the adjuvant chemotherapy trial of s-1 for gastric cancer (ACTS-GC), which was a randomized phase III trial in patients with stage II/III gastric cancer after curative surgery 5,7.

In this ACTS-GC trial, final outcomes (5-year relapse-free survival [RFS]) of patients with the 13th JGCA (Japanese Gastric Cancer Association) pathological stage (pStage) II, IIIA, and IIIB cancer were 79.2%, 61.4%, and 37.6% in the S-1 group and 64.4%, 50.0%, and 34.4% in the surgery alone group, respectively 7. A strong and satisfactory adjuvant effect of S-1 administration was observed, especially in gastric cancer patients with pStage II. However, even with the use of S-1, the clinical outcomes of patients with pStage III disease were unsatisfactory. We recently, explored prognostic factors in pStage III gastric cancer patients and identified the metastatic lymph node density (ND) as an independent prognostic factor. Moreover, patients with the 14th JGCA/7th UICC (Union for International Cancer Control) pStage IIIC gastric cancer in combination with high ND exhibited a dismal prognosis 8.

ND is defined as metastatic lymph ND and is expressed as a percentage of the number of metastatic lymph node against the number of dissected lymph node 8. ND may reflect the number of metastatic lymph nodes as well as the immune status of lymph nodes not involved with cancer further adjusting lymph node dissection level. It is, therefore, considered that ND is of high prognostic importance with clinical potential in esophageal 9,10 and gastric cancer 8,11–14. In the present study, we aimed to determine the mechanism that underlies the aggression of tumors with high ND, and to explore the expression of molecules associated with this important prognostic factor in order to develop a novel therapeutic strategy against clinically aggressive gastric cancer.

## Patients and Methods

### Registration of patients

Between 1 January 2000 and 31 December 2010, 1673 patients underwent gastrectomy for gastric adenocarcinoma in the gastrointestinal surgery division of Kitasato University Hospital. A total of 396 of these patients were diagnosed as the 13th JGCA stage II/III gastric cancer and underwent gastrectomy with D1+ or D2 lymph node dissection. Sixty seven of these 396 patients underwent neoadjuvant chemotherapy or postoperative chemotherapy other than S-1. Of the remaining 329 patients, 172 patients underwent adjuvant S-1 chemotherapy after surgery (S-1 standard treatment). We investigated 167 of the 172 patients who agreed to the use of their pathological specimens in this study.

### Clinicopathological factors

All histological and clinicopathological factors were assessed independently and blindly by histopathologists. Lymphatic permeation (ly) and vascular permeation (v) were defined as ly0, 1, 2, and 3, and v0, 1, 2, and 3 by infiltrative grade, but we classified ly and v as absence (ly0 and v0) or presence (ly1/2/3 and v1/2/3). Histologically, there are two major types of gastric adenocarcinoma (Lauren's classification). In this study, we classified cancers into diffuse type (por1, por2, sig, and muc) and intestinal type (pap, tub1, and tub2). ND has been defined as metastatic lymph ND against the dissected lymph node number 8.

### Microarray gene expression analysis

Total RNA from the primary gastric cancers of eight patients who had never undergone chemotherapy treatment before surgery was used to prepare biotinylated target cRNA according to the manufacturer's recommendations (provided by Affymetrix, Santa Clara, CA). The eight tumors were collected from the consecutively resected gastric cancers (*n* = 20) with UICC Stage III with outstanding contrast with regard to lymph node metastasis status from October 2008 to April 2009. Briefly, 200 ng of mRNA was used to generate first-strand cDNA using a T7-linked oligo-(dT) primer. After second-strand synthesis, the cDNA was subjected to in vitro transcription in the presence of biotinylated uridine triphosphate, using an IVT labeling kit (Affymetrix). Quantitative analyses of the isolated total RNA and the synthesized cRNA were conducted using electropherograms (Experion; Bio-Rad Laboratories, Hercules, CA). The biotinylated cRNA was fragmented and hybridized for 16 h at 45°C with the Human Genome U133 plus 2.0 array (Affymetrix), which contains an oligonucleotide probe set for 54,675 full-length transcripts and expressed sequence tags. The arrays were washed, stained with streptavidin-phycoerythrin, and scanned using the Affymetrix Model Fluidics Station 450 and the GeneChip Scanner 3000 (Affymetrix). The fluorescence intensity of each probe was quantified using the computer program GeneChip operating software, GCOS version 1.4 (Affymetrix). Each microarray was subjected to a standard quality control evaluation; the percentage of probe sets reliably detected (present flag) in each array was between 39.4% and 54.8%, and the 3′/5′ ratios for GAPDH and 18S rRNA gene were less than 5.25 and 2.19, respectively. BioB spike controls were also present on all the chips, with BioC, BioD, and Cre present in increasing intensities. *BioB, BioC, BioD,* and *Cre* are genes from *Escherichia coli* or bacteriophage P1 and were added before hybridization to check the hybridization quality. All background intensities and noise factors were within the acceptable range of 47.35–62.92 and 1.69–2.82, respectively.

### RNA purification and reverse transcriptase-polymerase chain reaction

Each sample of excised gastric tissue was immediately immersed in RNAlater RNA stabilization Reagent (QIAGEN Sciences, Maryland, MD), and the samples were homogenized for 60 sec at 2400 g using a MagNA Lyser (Roche diagnostics Inc., Mannheim, Germany). Harvested cells were washed three times with phosphate-buffered saline (PBS) and homogenized using QIA Shredder (QIAGEN). Total RNA from homogenized tissues and cell lines was extracted using the RNeasy Mini Kit (QIAGEN) and was reverse-transcribed using a SuperScript III Reverse Transcriptase kit (Invitrogen, Carlsbad, CA). Reverse transcriptase-PCR (RT-PCR) was performed. The PCR products were separated on 1.5–2.0% agarose gel, and visualized by ethidium bromide staining. Details of the PCR conditions and the sequences of the primers and probes used are shown in Table S1.

### Immunohistochemical staining of the EGFR

The primary antibodies used for immunohistochemical (IHC) assays were the mouse monoclonal antibodies against the human epidermal growth factor receptor (EGFR) that are included in the EGFR pharmDx kit (Dako-Japan, Tokyo, Japan). Routine formalin-fixed, paraffin-embedded tissue samples obtained from resected gastric cancer specimens were analyzed. Sections (3-*μ*m thick) were cut from the paraffin blocks and mounted on silanized slides. Immunohistochemical staining of the EGFR was performed using the EGFR pharmDx kit according to the manufacturer's instructions. The sections were deparaffinized in xylene and dehydrated using a graded ethanol series. After washing with distilled water, the sections were placed in the supplied buffer. For antigen retrieval, the slides were heated at 95°C for 40 min and then cooled for 20 min at room temperature. After washing with distilled water and with Tris-buffered 0.9% NaCl solution containing Tween 20 (pH 7.6), tissue sections were covered for 5 min with the peroxidase blocking reagent of the kit, followed by an additional washing with the supplied buffer. Individual slides were incubated for 30 min at room temperature with anti-EGFR antibody. The slides were then washed three times with the buffer and incubated with the polymer reagent of the kit for 30 min at room temperature. After extensive washing with PBS, the color reaction was developed using the DAB liquid system of the pharmDx kit for 6 min. The sections were then counterstained with hematoxylin, dehydrated, and mounted. We used a thing dyed from a specimen well every time as positive control.

### Scoring system of immunohistochemical staining

Cell membrane EGFR staining was assessed microscopically. All slides were blinded with regards to the prognostic analysis data. EGFR expression was graded using a 3-point scale, where 1+ = light staining of more than 10% of the specimens, 2+ = moderate staining of more than 10% and less than or equal to 30% of the specimens, 3+ = strong staining of more than 30% of the specimens. This scale was determined based on the diagnostic criteria of the American Society of Clinical Oncology/College of American Pathology 2007 guidelines 15.

### Statistical analysis

Cumulative 5-year RFS was estimated by the Kaplan–Meier method, and statistical differences were analyzed using the log rank test. RFS was measured from the date of surgery to that of recurrence or the last follow-up. The median observed terms were 24 months (from 3 to 107 months). A value of *P *<* *0.05 was considered statistically significant. The association between ND and clinicopathological factors or treatment factors was calculated using the least squares method. Factors showing *P *<* *0.10 by univariate analysis were subjected to multivariate analysis using a Cox proportional hazards model to identify independent prognostic factors. All statistical analyses were performed using SAS software package Stat View, version 5.0 (SAS Institute, Cary, NC) and/or JMP, version 10.0 (SAS Institute).

## Results

### Microarray analysis for the selection of candidate genes associated with high ND in pStage III gastric cancer

**Figure 1:**
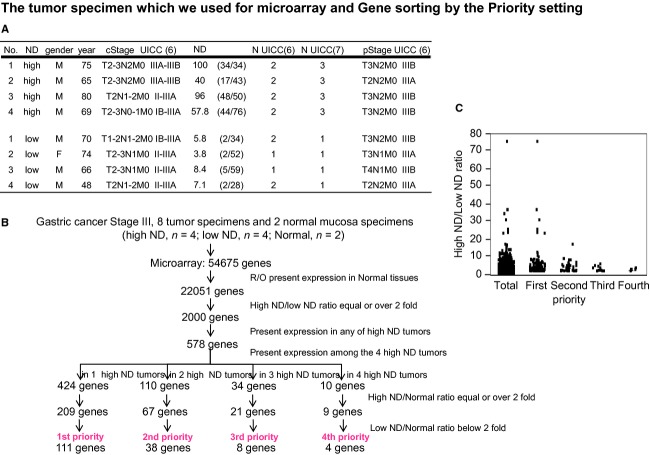
Gene sorting based on the priority settings and distribution. (A) The cases of high ND and low ND, which were selected from gastric cancer specimens. (B) Flowchart of the selection process of candidate genes associated with high ND in gastric cancer. (C) Distribution of high ND/low ND ratio values in each priority group.

To screen for candidate genes associated with high ND, cRNA was obtained from tumor specimens of eight patients with gastric cancer with 13th pStage III; (high ND, *n* = 4; low ND, *n* = 4, Fig.1A) and from two normal mucosa specimens, followed by cRNA hybridization to a 54,675 oligonucleotide microarray (Fig.1B). Each microarray was subjected to a standard quality control evaluation; the percentage of probe sets reliably detecting (present flag) was between 41 and 45%, and the 3′/5′ ratio of GAPDH was <1.25. All background intensities and noise factors were within the range of 40.10–48.40 and 1.20–1.70, respectively. Candidate high ND-associated genes were selected as follows (Fig.1B). The 32,624 genes obtained that were present in normal tissues were ruled out, following which, the 2000 genes that showed a high ND/low ND ratio equal to, or greater than twofold were identified. The number of candidates was further narrowed down by selection of genes that were expressed only in high ND tumors (578 genes). These genes were classified based on how many of the four high ND tumors expressed them. Of this group of genes, genes that displayed a high ND/normal ratio equal to or greater than twofold were further selected. Finally, prioritized genes that showed both a high ND/low ND ratio >twofold and a low ND/normal ratio below twofold were identified (1st priority = 111 genes, 2nd priority = 38, 3rd priority = 8, 4th priority = 4). The 1st priority genes represent both present and high expression in 1 tumors from the 4 cases. The 2nd priority genes represent both present and high expression in the 2 tumors. The 3rd priority genes were both present and high expression in the 3 tumors. The 4th priority genes were present and high expression in the 4 tumors.

Interestingly, the values of the high ND/low ND ratios in each priority group tended toward with distributed for high to low values according to the priority's number (Fig.1C, Table S2). Most of the molecules that were identified as first priority were genes such as *NKX2.1* 16,17, *CHST9* 18, *FGFR2* 19, *CTNND2* 20, and *EGFR* 21 that have been previously reported to show genomic amplification and overexpression in human cancer (Table S2).

### Validation of microarray results

**Figure 2:**
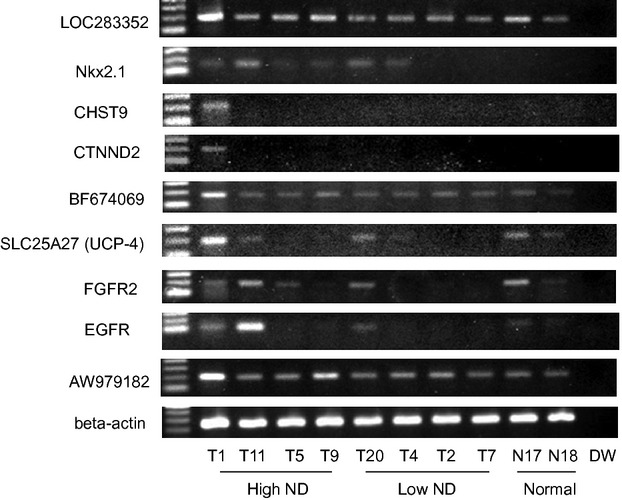
RT-PCR analysis of mRNA expression of the first priority genes.

Since the top ten genes of the 1st priority group included the above-described genes that show genomic amplification and/or overexpression in cancer, we considered that the 1st priority group of genes might be the highest and the most important priority group. We, therefore, validated the first priority genes by RT-PCR analysis of their expression in the four high- and four low-ND tumor tissues (Fig.2, Table S1).

*NKX2.1, SLC25A27, FGFR2, and EGFR* were highly expressed in high-ND tumors, and were especially highly expressed in one of these tumors, but were weakly expressed or barely detected in low-ND tumors. *CHST9* and *CTNND2* were highly expressed in one high-ND specimen but were not expressed in the three other high-ND tumors, the 4 low-ND tumors, or in the normal specimen. *LOC283352, BF674069,* and *AW979182* were constitutively expressed in all the 10 specimens. Of these gene candidates, we further focused on the association between *EGFR* and *NKX2.1*, and *EGFR* was ultimately proved to be associated with high ND.

### Clinicopathological analysis including EGFR status of pStage II/III advanced gastric cancer

**Figure 3:**
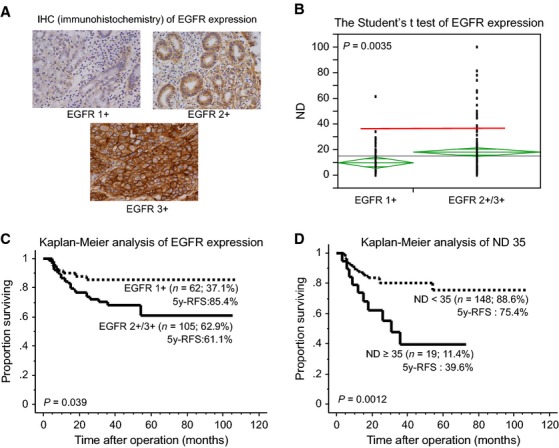
High EGFR expression is a strongly associated with high ND. (A) Microscopic analysis of cell membrane EGFR immunohistochemical staining. EGFR expression was graded using a 3-point scale, where 1+ = light staining of more than 10% of the specimens, 2+ = moderate staining of more than 10% and less than or equal to 30% of the specimens, and 3+ = strong staining of more than 30% of the specimens. (B) Statistical analysis of EGFR expression using Student's *t*-test. EGFR 2+/3+ expression group included more patients with high ND than the EGFR 1+ group. The most suitable ND cutoff level was deemed ND of 35%. (c) Kaplan–Meier curves indicate that EGFR 2+/3+ expression was significantly associated with poor outcome in patients with 13th JGCA stage II/III disease (*P *=* *0.039). (D) ND ≥35 was significantly associated with poor outcome (*P *=* *0.0012).

Since the assessment of EGFR expression using IHC is a well-established method, we used IHC to analyze EGFR expression in tumors from 167 gastric cancer patients with varying levels of ND after microarray experiments (Fig.3A). Statistical analysis of differences in EGFR expression between high- and low-ND tumors was determined using Student's *t*-test (Fig.3B). Between EGFR 1+ and 2+/3+, the widest margin of the distribution was recognized at the ND 35%. Analysis of both EGFR and clinicopathological features using Pearson's chi-square test is shown in Table1. Analysis of ND and clinicopathological features using Pearson's chi-square test is shown in Table2. ND greater than or equal to 35% were included in the EGFR 2+/3+ group (*P *=* *0.0023).

**Table 1:** Distribution of clinical and pathological factors of correlation with EGFR and univariate prognostic analysis in 167 pStage II/III gastric cancer with gastrectomy and subsequent S-1 treatment.

Variable	EGFR 1+ *n* (%)	EGFR 2+/3+ *n* (%)	*P-*value	5-year RFS (%)	*P-*value
Sex					
Male	39 (23.3)	78 (46.7)	0.12	60.0	0.030
Female	23 (13.8)	27 (16.2)		86.7	
Age (year)					
<67	38 (22.8)	58 (34.7)	0.44	79.4	0.0080
≥67	24 (14.4)	47 (28.1)		44.7	
Tumor location					
Upper	16 (9.6)	37 (22.1)	0.22	52.8	0.17
Middle	32 (19.2)	40 (23.9)		77.8	
Lower	14 (8.4)	28 (16.8)		81.2	
Lauren's histology					
Diffuse type	43 (25.7)	67 (40.1)	0.47	77.6	0.077
Intestinal type	19 (11.4)	38 (22.8)		51.0	
pT factor (13th JGCA)					
T2	19 (11.4)	43 (25.7)	0.40	96.7	0.023
T3	42 (25.2)	61 (36.5)		62.8	
T4	1 (0.6)	1 (0.6)		50.0	
pN factor (13th JGCA)					
N0	10 (6.0)	14 (8.4)	0.036	63.8	0.0030
N1	36 (21.6)	43 (25.7)		82.1	
N2	16 (9.6)	48 (28.7)		59.2	
pStage (13th JGCA)					
II	19 (11.4)	37 (22.1)	0.012	81.1	<0.0001
IIIA	36 (21.6)	39 (23.3)		74.1	
IIIB	7 (4.2)	29 (17.4)		47.1	
Infiltration pattern					
*α*	2 (1.2)	10 (6.0)	0.11	70.7	0.96
*β*	24 (14.4)	49 (29.3)		65.1	
*γ*	36 (21.6)	46 (27.5)		72.9	
Lymphatic permeation					
Yes	57 (34.1)	101 (60.5)	0.24	100.0	0.15
No	5 (3.0)	4 (2.4)		68.9	
Vascular permeation					
Yes	53 (31.7)	99 (59.3)	0.055	93.3	0.11
No	9 (5.4)	6 (3.6)		65.7	
ND					
ND < 35	61 (36.5)	87 (52.1)	0.0023	75.4	0.0012
ND ≥ 35	1 (0.6)	18 (10.8)		39.6	

**Table 2:** Distribution of clinical and pathological factors of correlation with ND in 167 pStage II/III gastric cancer with gastrectomy and subsequent S-1 treatment.

Variable	ND < 35 *n* (%)	ND ≥ 35 *n* (%)	*P-*value
Sex			
Male	103 (61.7)	14 (8.4)	0.71
Female	45 (26.9)	5 (3.0)	
Age (year)			
<67	85 (50.9)	11 (6.6)	0.97
≥67	63 (37.7)	8 (4.8)	
Tumor location			
Upper	46 (27.5)	7 (4.2)	0.60
Middle	63 (37.7)	9 (5.4)	
Lower	39 (23.4)	3 (1.8)	
Lauren's histology			
Diffuse type	96 (57.5)	14 (8.4)	0.45
Intestinal type	52 (31.1)	5 (3.0)	
pT factor (13th JGCA)			
T2	57 (34.1)	5 (3.0)	0.49
T3	89 (53.3)	14 (8.4)	
T4	2 (1.2)	0 (0)	
pN factor (13th JGCA)			
N0	24 (14.4)	0 (0)	<0.0001
N1	76 (45.5)	3 (1.8)	
N2	48 (28.7)	16 (9.6)	
pStage (13th JGCA)			
II	56 (33.5)	0 (0)	<0.0001
IIIA	67 (40.1)	8 (4.8)	
IIIB	25 (15.0)	11 (6.6)	
Infiltration pattern			
*α*	10 (6.0)	2 (1.2)	0.26
*β*	68 (40.7)	5 (3.0)	
*γ*	70 (41.9)	12 (7.2)	
Lymphatic permeation			
Yes	139 (83.2)	19 (11.4)	0.27
No	9 (5.4)	0 (0)	
Vascular permeation			
Yes	135 (80.8)	17 (10.2)	0.80
No	13 (7.8)	2 (1.2)	
EGFR			
1+	61 (36.5)	1 (0.6)	0.0023
2+/3+	87 (52.1)	18 (10.8)	

Clinicopathological features and prognosis (5-year RFS) were then analyzed in a univariate manner. Clinically significant potential prognostic factors representing poor survival in the S-1 group included male sex (*P *=* *0.030), age ≥67 years (*P *=* *0.0080), the 13th JGCA pT factor (*P *=* *0.023), the 13th JGCA pN factor (*P *=* *0.0030), the 13th JGCA stage (*P *<* *0.0001), and ND greater than or equal to 35% (*P *=* *0.0012). Log rank plot analysis indicated that the optimal cutoff values were defined for age ≥67 years 8.

Kaplan–Meier curves analyzing the association between EGFR expression and survival are shown in Figure3C. A case of EGFR 2+/3+ was significantly associated with poor outcome for 13th JGCA stage II/III disease (*P *=* *0.039). Kaplan–Meier curves of ND 35% are shown in Figure3D. ND ≥35% was a strong poor prognostic factor (*P *=* *0.0012).

According to the least squares method of analysis, when the ND score was above 35%, the risk that the EGFR expression score was IHC 2+ rather than IHC 1+ was 8.19 times higher for 13th JGCA pStage II/III gastric cancers treated with S-1 (*P *=* *0.0053) (Table3).

**Table 3:** The association between ND and clinicopathologic factors of the 13th JGCA pStage II/III gastric cancer using the least squares method.

Variable	Estimator	SD	*t-*value	*P-*value (Prob>|t|)	Lower 95% CI	Upper 95% CI
Graft	2.18	10	0.21	0.83	−18.25	22.6
EGFR [IHC2+ − IHC1+]	8.19	2.90	2.83	0.0053	2.47	13.91
EGFR [IHC3+ − IHC2+]	−3.72	3.2	−1.17	0.25	−10.02	2.58
Sex [Famale]	−1.03	1.4	−0.74	0.46	−3.77	1.71
Age	−0.06	0.1	−0.50	0.62	−0.30	0.18
Tumor location [L]	−0.55	1.8	−0.30	0.76	−4.19	3.08
Tumor location [M]	1.40	1.6	0.87	0.39	−1.78	4.57
Lauren's histology [diffused type]	0.14	1.4	0.10	0.92	−2.66	2.93
pStage(13th JGCA) [IIIA − II]	11.22	2.8	3.95	0.00010	5.61	16.84
pStage(13th JGCA) [IIIB − IIIA]	7.97	3.3	2.39	0.018	1.37	14.57
Infiltration pattern	−1.08	2.10	−0.52	0.61	−5.22	3.06
Lymphatic permeation	5.48	1.5	3.63	0.00040	2.50	8.47
Vascular permeation	−2.80	1.5	−1.93	0.055	−5.67	0.064

*R*^2^ = 0.36, RMSE = 14.8.

Based on a multivariate Cox proportional hazards model of factors associated with relapse-free survival of 13th JGCA pStage II/III gastric cancers, age (≥67) was a prognostic factor independent of the pStage (13th JGCA) (Table S3). ND and EGFR were not finally remnant in the multivariate analysis due to the small number of tumors that we tested. However, the primary endpoint of our study was to identify molecules associated with high ND rather than to determine the association of ND and EGFR with RFS.

## Discussion

In the present study, we compared gene expression in primary stage III gastric cancer tumors with high and low ND. We identified molecules whose expression is closely associated with high ND in gastric cancer and we confirmed a correlation between EGFR expression and high ND. Most of the molecules identified as top priority in the microarray analysis were genes that have been reported to be genomically amplified and overexpressed in human cancer. These genes were *NKX2.1* 16,17, *CHST9* 18, *FGFR2* 19, *CTNND2* 20, and *EGFR* 21. Three of the other top priority genes were *LOC283352*, *BF674069,* and *AW979182* whose expression in human cancers has not been previously reported.

The priority levels in the present study were based on the ratio of gene expression in 1, 2, 3, or 4 of the 4 tumors in the group. The 1st priority was considered as the highest or the most important priority because the average T/N ratio was much higher than that for the 2nd to the 4th priority. Moreover, the gene list for the 1st priority group included many already described oncogenes that are well known to be genomically amplified in cancer and that are bona fide therapeutic targets of cancer 22. However, it could be argued that, in contrast to our speculation above, the genes in the 4th priority group might be the most important set of genes, because the 4th priority group could represent the most consistent differences between ND groups across the set of tumors. We plan to determine the relevance of the 4th priority group of genes to ND in gastric cancer in the near future.

We were first focused on EGFR, because it was recently identified by the biomarker study after ACTS-GC trial; EGFR was proven to be a prognostic marker in stage II/III gastric cancer patients who underwent postoperative S-1 adjuvant therapy differently from HER2 23 as well as previous other study 24. Our immunohistochemical analysis of EGFR and HER2 also showed the similar those result, that EGFR expression had prognostic relevance for gastric cancer patients who underwent standard treatment (Fig.3C). In our study, EGFR expression was closely associated with extensive lymph node metastasis with high ND, suggesting that the EGFR might be a causative molecule for such aggressive phenotypes. Since pathological stage II/III gastric cancer is diagnosed after operation, postoperative adjuvant administration of anti-EGFR antibody in addition to the standard therapy may be a promising strategy for such gastric cancer.

In colorectal cancer, the *K-RAS* gene is used in practice as a biomarker for predicting the therapeutic effect of the anti-EGFR monoclonal antibodies cetuximab and panitumumab 25. Although we have not analyzed the genetic status of *K-RAS* in the current study, *K-RAS* mutations are not believed to be frequent in gastric cancer, and *K-RAS* genes are considered to be passenger genes during gastric cancer promotion 26. These studies suggest that gastric cancer patients with EGFR overexpression harbor the wild type *K-RAS* gene, and therefore could be promising for EGFR-targeted therapy. On the other hand, *EGFR* genomic amplification has been suggested to be an alternate and useful biomarker for predicting the therapeutic effect of an anti-EGFR monoclonal antibody in colorectal cancer 27. In this respect, it is interesting that *EGFR* genomic amplification has been previously reported in gastric cancer 28. We would, therefore, like to explore the clinical significance of EGFR genomic status in gastric cancer with ND in the near future.

The EGFR has already been demonstrated to be a good target of molecular therapy against gastric cancer. Thus, antisense inhibition of EGFR expression results in dramatic growth inhibition of gastric cancer cell lines with EGFR overexpression 29. Moreover, EGFR inhibition by cetuximab, a monoclonal anti-EGFR antibody, synergized with a chemotherapeutic drug for antitumor activity against gastric cancer cell lines 30. In a clinical setting, when erlotinib, an oral EGFR inhibitor, was administered to metastatic or unresectable cases of gastro-esophageal (GE) junction and gastric adenocarcinomas in SWOG0127 trials 31, more cases of GE junction cancer than of gastric cancer responded clinically. Furthermore, a combination of mFOLFOX6 and erlotinib treatment proved to be a feasible and active treatment for patients with GE junction tumors, in a phase II clinical trial 32.

ND is defined as metastatic lymph node density against the dissected lymph node number 33–35. In the present study, ND of greater than 35% (ND 35%) was a strong prognostic factor in gastric cancer, which is consistent with the conclusions of our previous studies (Fig.3D) 35,36. However, perhaps the most interesting result of the present study is that ND 35% showed significant association with high expression of the *EGFR* gene (Fig.3B). High *EGFR* expression might thus be the causative mechanism of the aggressiveness of gastric cancer with high ND. EGFR inhibition may, therefore, prove to be the optimal target for therapy of gastric cancer with high ND, when used as a postoperative adjuvant therapy.

However, a role for other genes in the aggressiveness of high ND in gastric cancer cannot be excluded. In this respect, we considered that *NKX2.1* was the second-most interesting gene of the genes that we identified in our screen, because this was the first demonstration of a considerable number of *NKX2.1* genomic amplifications in gastric cancer (Fig. S1A). *NKX2.1* has recently been proposed to be a critical oncogene for, and genomically amplified in lung cancer 16,17. However, we found much lower expression of *NKX2.1* in gastric cancer cell lines than in lung cancer cell lines by Western blotting (Fig. S1B and C). Using immunohistochemical staining, we also found that *NKX2.1* was predominantly expressed in peritoneal dissemination, but not in primary gastric cancer. *NKX2.1* induces the expression of HOP homeobox (*HOPX*) in lung cancer 37. *HOPX* is a strong tumor suppressor gene that was identified using pharmacological unmasking microarrays 38–42. In contrast to the situation in lung cancer, in gastric cancer, the promoter CpG islands of the *HOPX* gene were found to be strongly hyper-methylated and *HOPX* gene expression was silenced differently from lung cancer 37,41. For these reasons, lower levels of *NKX2.1* may be more effective as an oncogene in gastric cancer than in lung cancer. A recent study also demonstrated that NKX2.1 is required to sustain EGFR survival signaling in human cancer 43. Consistent with that result, our data (Fig.2) showed that *NKX2.1* expression was closely associated with EGFR expression, and that expression of either molecule in primary gastric cancer may represent activation of the same pathway.

Of the other genes that we identified in the 1st priority group, CHST9 is a sulfo-transferase and the *CHST9* gene was recently shown to be amplified in multiple types of hematological malignancies 18. Additionally, specific sulfated glycosaminoglycans (GAGs) were recently shown to be involved in lung metastasis, and such lung metastasis could be suppressed by specific antagonists 44. Gastric cancer sometimes metastasizes to lung, and therefore it would be interesting to determine if CHST9 plays a causative role in the formation of such GAGs in the lung metastasis of gastric cancer. CTNND2 has been designated as δ-catenin. *CTNND2* is overexpressed in human cancer, and exogenous *CTNND2* overexpression promotes a malignant phenotype 45. *CTNND2* genomic amplification and genetic changes have also been reported in cervical cancer 20 and prostate cancer 46, respectively. Thus, *CTNND2* is also a promising target for therapy of gastric cancer. Finally, *FGFR2* is well known as an alternate oncogene that can be molecularly targeted in gastric cancer 47,48. A recent comprehensive survey of genomic alterations in gastric cancer identified change in *FGFR2* expression as the most frequent change among the RTK (receptor tyrosine kinase)/*RAS* family genes 49. Even in our initial screening (Fig.2), *FGFR2* expression, similar to that of *NKX2.1* correlates well with that of *EGFR* expression in primary gastric cancer (Fig.2). In order to determine the optimal therapeutic target for future studies, the question that needs to be resolved is which of *EGFR, FGFR2,* and *NKX2.1* is the most upstream and the critical alteration in primary gastric cancer.

In conclusion, we identified the *EGFR* gene as the reemerged molecule which explains the mechanism of aggression of advanced gastric cancer with high ND. The EGFR may be one of the most optimal molecular targets in therapy of aggressive gastric cancer, which is resistant to the current standard chemotherapy regimens. Further analyses are required to elucidate the most upstream causative gene of gastric cancer.

## Supporting Information

**Figure S1.** Gene amplification and expression of *NKX2.1* in gastric cancer. (A) The frequency of *NKX2.1* gene amplification in 13th pStage I to III gastric cancer (GC) (IA, *n* = 15; EGC IB, *n* = 15; AGC IB, *n* = 20; III low ND, *n* = 35, III high ND, *n* = 17). Genomic DNA from FFPE tissue was extracted using QIAamp DNA FFPE Tissue kit (QIAGEN) according to the manufacture's protocol. To examine the copy number of the genes, quantitative-PCR (Q-PCR) was performed in triplicate, and we defined that over twofold copy number was gene amplification. (B) Expression of *NKX2.1* by western blotting (top panel) and RT-PCR (bottom panel) in GC cell lines. Lung cancer cell line NCI-H661 was used as a positive control. Cell lysates were prepared and were analyzed by western blotting using an anti-NKX2.1 mouse IgG_1_/Kappa monoclonal antibody (dilution of 1:5000, Seven Hills Bioreagents, Cincinnati, OH) and an anti-beta-actin mouse IgG_2a_ monoclonal antibody (dilution of 1:10,000, Sigma-Aldorich Inc., St. Louis, MO). (c) Immunohistochemical staining using mouse monoclonal IgG_1_ NKX2.1 antibody (dilution of 1:100, Invitrogen, Carlsbad, CA) in primary tumor and peritoneal dissemination. Normal thyroid tissue was immunostained as a positive control. Original magnification, X200. Scale Bars, 100 *μ*m.

**Table S1.** PCR production and sequence of primers and fluorescent probe.

**Table S2.** Candidate genes in each priority.

**Table S3.** Multivariate analysis of factors associated with relapse-free survival of the 13th JGCA pStage II/III gastric cancer.
