# Supplementary material for: Identification of EGFR expression status association with metastatic lymph node density (ND) by expression microarray analysis of advanced gastric cancer
Source: Cancer Med. 2014 Aug 26;4(1):90–100. doi: 10.1002/cam4.311 (PMC4312122; doi:10.1002/cam4.311)
Supplement: Supplementary file 1 [file cam40004-0090-sd1.ppt]

## Slide 1
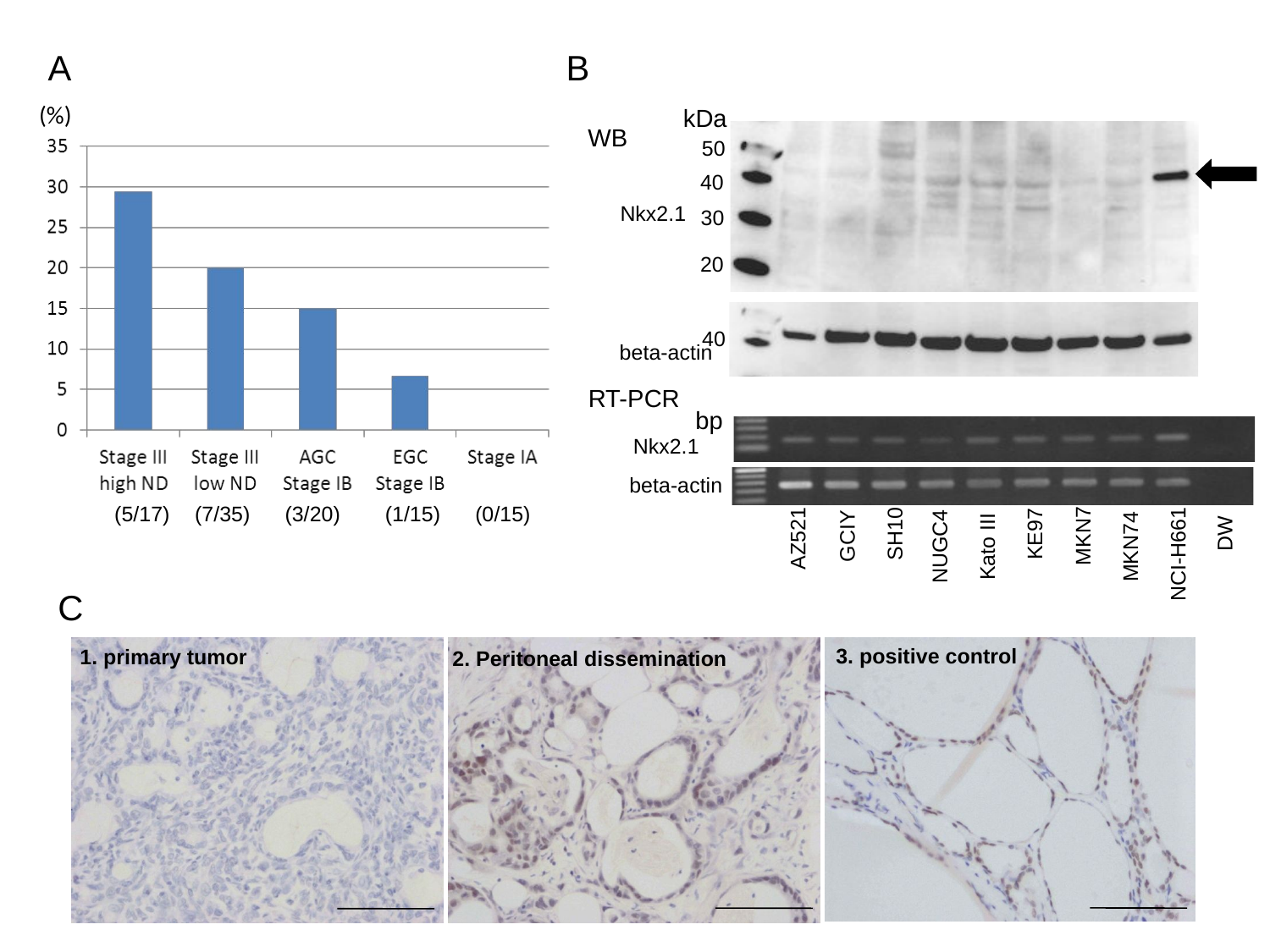

A
(%)
(1/15)
(0/15)
(5/17)
(7/35)
(3/20)
 B
kDa
Nkx2.1
50
40
30
20
WB
40
beta-actin
RT-PCR
bp
Nkx2.1
SH10
KE97
DW
GCIY
MKN7
AZ521
NUGC4
Kato III
MKN74
NCI-H661
beta-actin
C
3. positive control
1. primary tumor
2. Peritoneal dissemination
2. primary tumor
